# Evaluation of Platelet Indices and Reticulated Platelets Using the ADVIA 2120 Analyzer in Patients with Acute Infection or Acute Coronary Syndrome, at Onset

**DOI:** 10.3390/medsci13040232

**Published:** 2025-10-14

**Authors:** Vincenzo Brescia, Antonella Mileti, Roberto Lovero, Lucia Varraso, Francesco Pignataro, Francesca Di Serio, Angela Pia Cazzolla, Luigi Santacroce, Maria Eleonora Bizzoca, Vito Crincoli, Maria Severa Di Comite

**Affiliations:** 1 Clinical Pathology Unit, AOU Policlinico Consorziale di Bari—Ospedale Giovanni XXIII, 70124 Bari, Italy; bresciavincenzo58@gmail.com (V.B.); antonellamileti@libero.it (A.M.); robertolovero69@gmail.com (R.L.); lu.varraso@gmail.com (L.V.); francescopignataro1990@gmail.com (F.P.); francesca.diserio@policlinico.ba.it (F.D.S.); 2 Department of Clinical and Experimental Medicine, Università degli Studi di Foggia, 71122 Foggia, Italy; mariaeleonora.bizzoca@unifg.it; 3 Microbiology and Virology Lab., Ionian Department, Policlinico University Hospital, University of Bari “Aldo Moro”, Piazza G. Cesare 11, 70124 Bari, Italy; luigi.santacroce@uniba.it; 4 Interdisciplinary Department of Medicine, University of Bari “Aldo Moro”, Piazza G. Cesare 11, 70124 Bari, Italy; vito.crincoli@uniba.it; 5 Department of Translational Biomedicine and Neuroscience (DiBraiN), University of Bari “Aldo Moro”, Piazza Giulio Cesare, 70124 Bari, Italy; mariasevera.dicomite@uniba.it

**Keywords:** platelet indices, hematology analyzers, sepsis, acute coronary syndromes

## Abstract

Background: The aim of this study was to evaluate the changes in platelet indices (PLT) provided by the ADVIA 2120 hematology analyzer (Siemens Hematology System) in the early stages of onset of infections and acute coronary syndromes (ACSs). Methods: Samples were selected from 40 patients admitted to the intensive care unit with suspected uncomplicated sepsis at presentation, from 40 patients with a biochemical diagnosis of ACS at presentation and from 40 apparently healthy subjects. These samples were tested for PLT and PLT indices [mean platelet volume (MPV); mean platelet mass (MPM); mean platelet component (MPC); immature platelets (RtcPlts)] obtained by automation with the ADVIA 2120 and specific biomarkers for sepsis [white blood cells (WBCs); neutrophil granulocytes (NGs); presepsin (PSP); procalcitonin (Pct); C-reactive protein (CRP)] and for SCA (hs cTnI). Results: Platelet indices (RtcPlts, MPV, MPM) were significantly altered (*p* > 0.005) in patients with suspected sepsis and patients with ACS compared to control subjects; however, no statistically significant difference was observed between the two groups of patients with disease. Cutoff values (ROC curves) were obtained for platelet indices that best discriminated healthy subjects from subjects with severe infection or ACS. Conclusions: Our data show that, in subjects with suspected sepsis and ACS at disease onset, a state of early platelet activation exists that is not disease-specific. Immature platelets (RtcPlts) and the platelet indices MPM and MPV, provided by the ADVIA 2120 hematology analyzer, showed high sensitivity in subjects with suspected sepsis or ACS at disease onset.

## 1. Introduction

Platelets are multifunctional, disk-shaped cells with a diameter of 1 to 3 μm. Reticulated platelets (RPs) are hyper-reactive immature platelets newly released from the bone marrow; compared to mature platelets, RPs are larger and contain more RNA; they have higher concentrations of thrombotic mediators and greater ability to participate in thrombosis [1,2].

Ultrastructural analysis reveals that RPs contain a complete Golgi apparatus and a rough endoplasmic reticulum, absent in non-RP platelets, and are therefore capable of protein biosynthesis [3,4]. Platelets have a role that goes beyond being simple actors in hemostasis and thrombosis and are involved in inflammatory or immune processes. Upon activation, platelets release secretory products and express membrane immune receptors that participate in the recruitment of leukocytes into inflamed tissue [5]. Platelets are able to form aggregates with neutrophils (Platelet–Neutrophil Complexes, PNCs), which leads to the activation of both cells with consequent release of cytokines and exposure of some adhesion molecules that facilitate the arrival of cells in the inflamed tissue. The complex regulatory mechanism of the biology and physiopathology of platelet activation is also involved in the interleukins, a heterogeneous family of cytokines produced by various cells that play a crucial role in immune responses and immunoregulation [5]. The interleukins (IL6 and IL3) produced by platelets during inflammation facilitate the rupture of megakaryocytes and increase the levels of thrombopoietin (TPO), thus improving platelet production [6]. Much of our knowledge about cross-linked platelets comes from the observation of size heterogeneity, especially under conditions of stimulated thrombopoiesis. The RNA present in cross-linked platelets gradually degrades and the cell size decreases until they become mature elements; therefore, these immature platelets can be recognized in the peripheral blood for 1 or 2 days after release from the bone marrow [7]. Cross-linked platelets have been studied in several non-hematological pathological conditions, especially in the diagnosis and prognosis of cardiovascular diseases and as a biomarker for the early diagnosis and monitoring of sepsis in critically ill patients [8]. The evaluation and comparison of cross-linked platelets against indicators of inflammation or necrosis have improved the diagnostic sensitivity and specificity in the early onset phase of several diseases and has highlighted a potential prognostic value in multiple clinical settings [9]. Current automated hematology analyzers are able to provide parameters related to platelet morphology and proliferation kinetics, indicated as “platelet indices”. The available “platelet indices” are mean platelet volume (MPV), a measure calculated by the platelet volume analyzer and expressed in femtoliters (fL); mean platelet mass (MPM), a measure calculated from the platelet mass histogram and expressed in picograms (pg); and mean platelet component (MPC), a measure of the mean refractive index of platelets which correlates to the granule content and density of platelets, expressed in grams/deciliter (g/dL). These platelet indices are available on all analyzers, can be reported, are related to the presence of immature platelets and have been proposed as potential biomarkers in numerous diseases, both acute and chronic [10,11]. Hematology systems are able to distinguish a state of platelet activation and immaturity using flow cytometry and by studying cell size and intracellular density. The available parameters are (i) reticulated platelets (retPLT) provided by Abbott analyzers; (ii) immature platelet fraction (IPF) provided by Sysmex and Mindray analyzers; and (iii) RtcPlts (% immature platelets) provided by Siemens analyzers [12,13]. These assessments are method-dependent and show only modest correlations between them, and to date not all analyzers have validated cutoffs that allow widespread clinical use [10,14,15,16]. The aim of this study was to evaluate the performance and diagnostic accuracy of platelet maturation parameters and indices provided by the ADVIA 2120 hematology analyzer (Hematology System Siemens) in the early stages of infection onset and in acute coronary syndromes (ACSs).

## 2. Materials and Methods

### 2.1. Study Subjects

This prospective observational study was conducted in accordance with the principles of the Declaration of Helsinki and the International Conference on Harmonization Guidelines for Good Clinical Practice. Before the start of the study, ethical approval was obtained from the relevant institutional review committees of the Policlinico University Hospital of Bari (protocol number 0034687; date of approval: 12 May 2020). All patients received complete information on the nature and objectives of the study before signing a form for informed consent, and informed consent was obtained from all subjects involved in the study.

### 2.2. Inclusion Criteria

Forty patients admitted to the ICU at the University of Bari Hospital with infection and uncomplicated sepsis at presentation, defined by the presence of both infection and systemic inflammatory response syndrome (SIRS) without any evidence of organ dysfunction based on biochemical parameters, were recruited. The diagnosis of infection was made using SIRS (systemic inflammatory response syndrome) criteria (temperature > 38 °C or <36 °C, heart rate > 90/min, respiratory rate > 20/min or PaCO_2_ < 32 mm Hg (4.3 kPa), leukocyte count > 12,000/mm^3^ or <4000/mm^3^ or >10% immature bands [17]).

Furthermore, 40 patients with a biochemical diagnosis of ACS at presentation according to the guidelines were selected [18].

Apparently healthy individuals between the ages of 18 and 60 were enrolled. These subjects attended the Clinical Pathology operating unit for routine clinical monitoring.

The exclusion criteria were pregnancy, known endocrine/metabolic diseases, renal insufficiency (eGFR < 75 mL/min/1.73 m^2^), cancer, hospitalization in the last 4 months, biochemical signs of acute or chronic infection, stable ischemic heart disease, and presence of other causes of elevation of biomarkers of myocardial ischemic damage [18]. Samples with serum values of creatinine, azotemia, sodium, potassium, calcium, phosphate, glycemia, enzymes and liver function tests, TSH, FreeT4, blood cell count, albuminemia, iron, transferrin, ferritin or C-reactive protein which were within the reference limits adopted in the laboratory were considered normal and considered suitable.

### 2.3. Sample Collection

Blood samples were collected between December 2023 and January 2024. During the entire collection period, an individual subject was selected only once to provide a sample. A total of 120 selected samples consisted of those of 40 patients with suspected sepsis at presentation, 40 patients with ACS at presentation, and 40 healthy controls. An EDTAK3 whole blood sample was collected for complete blood count and a serum tube blood sample for clinical chemistry. Serum samples with potential interference from hemolysis (hemoglobin > 500 mg/dL) and chylosity (triglyceride concentrations > 1000 mg/dL) assessed using the HIL method [Dimension VISTA 1500 instrumentation (Siemens, Munich, Germany)] were excluded. All analyses were performed within three hours of collection.

### 2.4. Analytical Measurements

The procalcitonin (Pct) test (target value 0.0–0.05 ng/mL) (Elecsys BRAHMS PCT kit, Roche, Mannheim, Germany) was performed with the chemiluminescence test using Cobas e801 (Roche, Mannheim, Germany), the presepsin test (target value 20–200 pg/mL) (PATHFAST Presepsin (PSP) CARTRIDGE kit, Mitsubishi Chemical Europe GmbH, Düsseldorf, Germany) with the chemiluminescence test using PA-THFAST^®^ Presepsin, the nephelometric C-reactive protein (CRP) test (target value 0–3 mg/L) (SIEMENS CRP Flex reagent cartridge kit, Munich, Germany) using Siemens Dimension Vista 1500 (SIEMENS, Munich, Germany); and the Troponin I hs (hs cTnI) test (analytical sensitivity of 0.5 ng/L, a linear range of 3.0–25,000 ng/L, an intra-assay variation of 4.7% and inter-assay variation of 7.6%) with a LOCI chemiluminescence assay (using Siemens Dimension Vista 1500 (SIEMENS, Munich, Germany). For measurement of blood count parameters, samples were collected in tubes with EDTAK3 anticoagulant and analyzed on the ADVIA 2120 hematology analyzer (Hematology System Siemens). Reagents supplied by the manufacturer were used to determine the blood count parameters.

White blood cell (WBC) count (103/mL) and neutrophil granulocyte (GN%) percentage were obtained by flow cytometry with the peroxidase method. The platelet (PLT) study was performed by the optical method and the following parameters were considered: PLT (platelet count), ×103/µL; RtcPlts (%) (immature platelets)%; MPV (mean platelet volume calculated from a platelet volume histogram), fL; MPC (mean platelet component or granule concentration calculated from a platelet component histogram reflecting platelet density), g/dL; and MPM (platelet dry mass or mean granule content calculated from a platelet dry mass histogram), pg.

In particular, materials for Internal Quality Control (level low, intermediate or high) supplied by the manufacturing company were used and analyzed in each assay to monitor the analytical precision (CV%). Furthermore, the laboratory participated in the EQAS (External Quality Assessment Services) program to verify analytical accuracy.

### 2.5. Statistical Analysis

Age, sex, platelet parameters and concentration of biomarkers assessed in apparently healthy, infected and ACS subjects are reported using descriptive statistics for quantitative variables.

Mean and median concentrations and standard deviations (SDs), distribution ranges [0.05 and 0.95 percentiles and 95% confidence intervals (CIs)] of PLT × 10^3^/µL, RtcPlts (%) %, platelet indices (MPC g/dL, MPM pg/dL, MPV fL), WBC × 10^3^/µL, NG%, inflammation biomarkers (PSP pg/mL, CRP mg/L) and hs cTnI ng/L were calculated using standard parametric and non-parametric statistical analyses. Normality of distribution was assessed using the D’Agostino–Pearson test; the distribution was considered normal if the *p*-value was greater than 0.05.

To visualize the distribution of samples and the underlying normality curve, the frequency distribution histogram (%) was used.

Concentration boxplots were produced. The center box shows the 25th to 75th quartile values, the center line the median, and the horizontal lines the span from the minimum to the maximum value (range of the distribution for each biomarker) for a visual interpretation of the numerical data and to show the number of concentrations falling outside the specified range of values. The Mann–Whitney U test was used to compare whether the median biomarker concentrations, and platelet indices provided in automation between the control group and the group with suspected sepsis onset and between the control group and the group with ACS onset. Receiver operating characteristic (ROC) curves were used to calculate the cutoff values of platelet indices and RtcPlts (%) (immature platelets) % that best discriminate between the control group and patients with suspected sepsis and the control group and those with ACS.

The area under the ROC curve (AUC) provided a measure of the ability of each of the analytes included in the study to distinguish between the healthy group (H) and the disease groups. The AUC was classified as “excellent” for values between 0.9 and 1; “very good” between 0.8 and 0.9; “good” between 0.7 and 0.8; “fair” between 0.6 and 0.7; “sufficient” between 0.5 and 0.6 and “poor” at <0.5 (useless test) [19]. The accuracy of each biomarker was then calculated and compared following the application of the best cutoff values determined by the ROC curve analysis to derive the sensitivity and specificity. A p-value threshold of 5% was adopted for all tests used.

Summary tables have been produced to highlight the variations in concentration of the different markers used and the results of the statistical tests obtained.

All statistical analyses were performed using the MedCalc program v 9.2.0.2 (Med-Calc Software, Ostend, Belgium).

## 3. Results

The subjects included in the control group were 22 males (55%) (mean age 42 years) and 18 females (45%) (mean age 42 years); those in the group with suspected sepsis were 24 males (60%) (mean age 54 years) and 16 females (45%) (mean age 53 years); those in the group with ACS were 23 males (57%) (mean age 57 years) and 16 females (45%) (mean age 56 years). The distribution of platelets, MPC, MPM, MPV, leukocytes, GN, PSP, Pct, PCR and hs cTnI in healthy subjects is reported in Table 1 and was within the range of reference values used in the laboratory and included in the report.

The distribution of platelets, RtcPlts (%), MPC, MPM, MPV, leukocytes, neutrophil granulocytes %, PSP, Pct and PCR in subjects with suspected sepsis at onset is reported in Table 2.

White blood cell count (WBC), GN% values and concentration of suspected sepsis biomarkers (Pct, PSP, PCR) were strongly indicative of an ongoing infection; platelet indices RtcPlts (%), MPV and MPM showed median values higher than those of samples from apparently healthy subjects. The difference between the medians, evaluated with the Mann–Whitney test, was statistically significant (*p* < 0.05), indicating probable platelet activation in the early stages of the infection. Only the evaluation parameter MPC did not show a statistically significant difference (*p* > 0.05) (Table 3).

**Figure 1:**
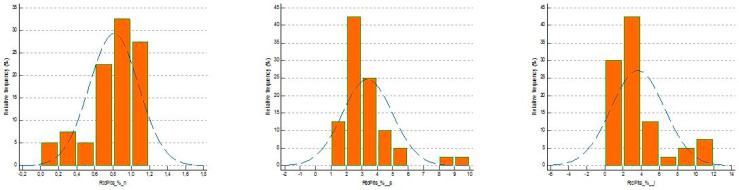
Histograms with frequency distributions of RtcPlt (%) in healthy subjects (n), in patients with suspected sepsis (s) at onset and in patients with ACS (i) at onset. The number of data points falling within a specified range of values and the normal distribution curve (with the mean and standard deviation of the data points represented in the histogram) are displayed.

**Figure 2:**
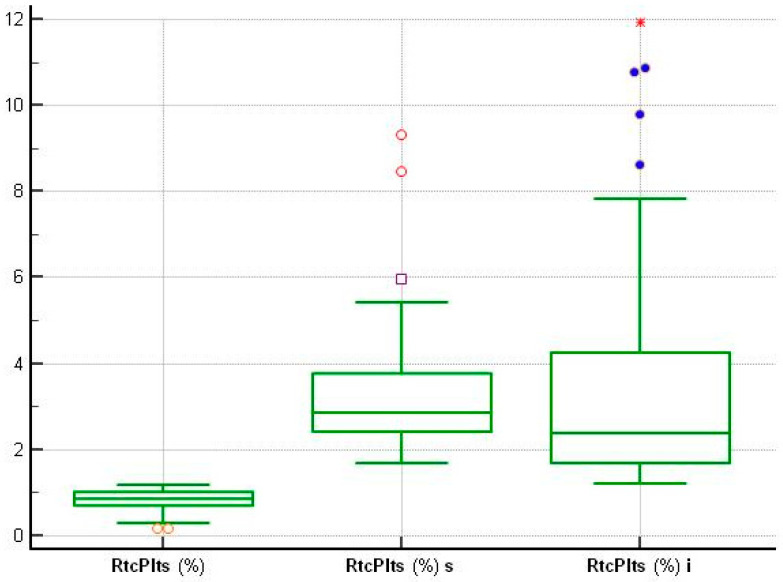
Boxplot showing the statistical summary of RtcPlt (%) in healthy subjects, patients with suspected sepsis (s) at onset and patients with ACS (i) at onset. The plot shows the values from the lower to the upper quantile (25th to 75th percentiles) in the central box, the median line, the vertical line extending from the minimum to the maximum value and the outer and far values, which are displayed as separate points.

The difference in the distribution of values was confirmed by the display of frequency distribution histograms (%) (Figure 1) and the boxplot of concentrations (Figure 2).

**Figure 3:**
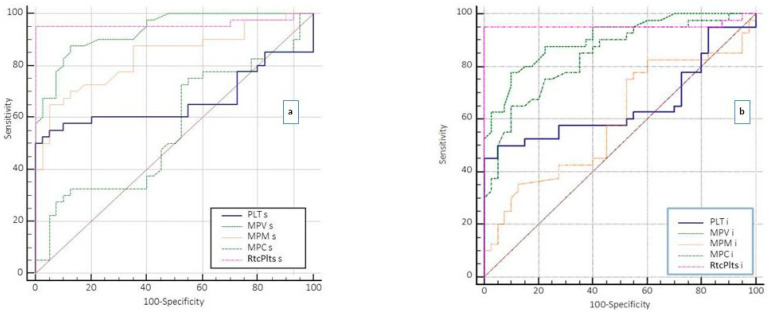
(**a**) Receiver operating characteristic (ROC) curve representing the true positive rate (sensitivity) versus the false positive rate (specificity 100) for different cutoffs of PLT, MPV, MPM, MPC and RtcPlt obtained in patients with suspected sepsis at presentation. The closer the ROC curve is to the upper left corner, the higher the diagnostic accuracy of the test. (**b**) Receiver operating characteristic (ROC) curve representing the true positive rate (sensitivity) versus the false positive rate (specificity 100) for different cutoffs of PLT(i), MPV(i), MPM(i), MPC(i) and RtcPlt(i) obtained in patients with ACS at presentation. The closer the ROC curve is to the upper left corner, the higher the diagnostic accuracy of the test.

In patients with suspected sepsis, the ROC curve analysis used to evaluate the degree of diagnostic accuracy based on the cutoff of platelet parameters showed excellent accuracy for RtcPlts (%) (AUC = 0.96); very good accuracy for MPV (AUC = 0.93) and MPM (AUC = 0.84); sufficient accuracy for PLT (AUC = 0.66); and poor accuracy for MPC (AUC = 0.56) (Figure 3a and Table 4).

Among all platelet parameters evaluated in subjects with suspected sepsis at onset, RtcPlts (%) had the best sensitivity vs. specificity ratio at the cutoff of 1.15 (Table 5).

The evaluation of the results obtained supports the hypothesis that during the initial phase of infection the most sensitive parameter for platelet activation is RtcPlts (%).

The distribution of PLT, RtcPlts (%), MPC, MPV, MPM and hs cTnI in subjects with suspected ACS is reported in Table 6. All hs cTnI values of patients included in this study with ACS were above 51.1 pg/mL, considered the 99th percentile cutoff value for AMI as indicated in the ESC guidelines. The platelet indices RtcPlts (%), MPV and MPM of subjects with ACS showed higher median values than those of samples from apparently healthy subjects with a statistically significant difference (*p* < 0.05) (Mann–Whitney U test), indicating early platelet activation (Table 7).

In patients with ACS, the analysis of the ROC curves of platelet parameters showed excellent accuracy for RtcPlts (%) (AUC = 0.95); very good accuracy for MPV (AUC = 0.90) and MPM (AUC = 0.84); sufficient accuracy for PLT (AUC = 0.66); and poor accuracy for MPC (AUC = 0.59) (Figure 3b and Table 8). To obtain a robust estimate of the performance on the unprovided data and to avoid overfitting in a small dataset, the confidence interval of the AUC was calculated and provided (Table 8). Among all platelet parameters assessed in subjects with ACS at onset, RtcPlts (%) had the best sensitivity vs. specificity ratio at the cutoff of 1.16 (Table 5).

The visualization of the frequency distribution histograms (%) (Figure 1) and the concentration boxplot (Figure 2) confirmed the difference in the distribution of RtcPlts (%) values in patients with ACS at onset compared to control subjects.

The Mann–Whitney U test for the comparison between the medians of patients with suspected sepsis at presentation and patients with ACS at presentation did not show a statistically significant difference (*p* > 0.05) between PLT, MPV, MPM, RtcPlts (%) and MPC (Table 9).

Therefore, platelets present an early activation phase, probably related to the rapid release of cytokines, without differences between the two clinical conditions.

## 4. Discussion

Reticulated platelets (RPs) are the youngest platelet population. Their presence is a useful indicator of platelet reactivity following thrombotic phenomena and nonspecific inflammatory processes accompanying various pathological conditions [20,21].

The immature platelet fraction has potential clinical applications in the diagnosis and monitoring of different diseases. Among the clinical applications, the early diagnosis of sepsis and acute coronary syndrome is certainly among the most studied [22,23].

Currently, the diagnosis of sepsis depends mainly on the clinical presentation, aided by the measurement of biochemical indicators of inflammation. Procalcitonin (Pct) and C-reactive protein (CRP) are currently the most commonly used laboratory biochemical parameters, although they lack specificity in systemic inflammatory response syndrome (SIRS) and do not allow an early diagnosis of sepsis [24,25,26].

Hyper-reactive immature platelets play a key role in the pathogenesis of sepsis as they cause predisposition to alterations of the microcirculation as a consequence of thrombotic phenomena. Increased levels of immature platelets have been found to be significantly correlated with the severity of the infection [12,27]. Immature platelets increase before the onset of sepsis with a prognostic efficiency equal to that of CRP. Our promising data are influenced by the small sample size resulting from the restrictive selection of subjects who presented a “severe infection” at onset not classifiable as sepsis [17] and the single-center design of the study [28,29].

Data from our preliminary study have highlighted that in the early stages of the infection the absolute number of platelets (PLT), platelet indices (MPV, MPM) and RtcPlts provided by the ADVIA 2120 hematology analyzer appear significantly modified, together with the generic indicators of inflammation [peripheral leukocyte count (WBC), percentage of neutrophils (NG%), C-reactive protein (CRP), procalcitonin (Pct), presepsin (PSP)]. In particular, we found that RtcPlts (%) and platelet index values were significantly higher than in healthy subjects in the control group. The sensitivity and specificity of RtcPlts (%) in detecting serious infections were 95% and 100%, respectively, at a concentration of 1.15 with excellent accuracy (AUC = 0.96; 95%CI 0.90 to 1.0).

Other authors have confirmed the diagnostic performance of immature platelet counting in its ability to discriminate septic from non-septic patients with an accuracy comparable to current infection biomarkers such as C-reactive protein and procalcitonin [12,30]. The variations in platelet indices and reticulated immature platelets (RtcPlts) obtained with Siemens ADVIA 2120 could be used as nonspecific biomarkers able to provide an additional element to identify patients with uncomplicated infections at onset [26]. The combination of biochemical markers, platelet indices and immature platelets could improve the sensitivity and specificity of early diagnosis of infections at onset in the time frame of our study, anticipating the possible positivity of blood cultures and the severity of organ damage by anticipating the specific clinical manifestations of sepsis and providing indications of the clinical course [31,32].

However, the immature platelet count (RtcPlts) obtained with the Siemens ADVIA 2120 analyzer needs to be further studied to confirm whether routine monitoring can be useful in recognizing the severity of sepsis and in predicting mortality in patients with sepsis and to confirm that the diagnostic accuracy is similar to that of other analytical systems [33,34,35].

Platelets are essential in the pathogenesis of all types of coronary artery disease, being involved in endothelial dysfunction, atherosclerotic lesions and thrombotic complications [36]. In patients with acute myocardial infarction [with ST-segment elevation (STEMI) or without ST-segment elevation (non-STEMI)] and unstable angina pectoris, immature platelet levels are elevated, with a significant increase in patients with acute coronary syndrome (ACS) compared to stable angina [37]. The percentage of immature platelets increases with increasing severity of ACS (10.53% in unstable angina, 15.99% in non-STEMI and 18.94% in STEMI) [38].

Data from our preliminary study show that in the early stages of ACS, the absolute platelet count (PLT), platelet indices (MPV, MPM) and RtcPlts provided by the ADVIA 2120 hematology analyzer appear significantly modified, together with hs Troponin I (hs cTnI) (mean and CI). The median values of RtcPlts (%) and platelet indices were significantly higher than in healthy subjects in the control group. The ROC curve analysis of our study determined that the sensitivity and specificity of RtcPlts (%) in detecting ACS at onset were 95% and 100%, respectively, at a concentration of 1.16 with excellent accuracy (AUC = 0.95; 95% CI 0.89 to 1.0). In the literature, RP levels are reported to be elevated not only in sepsis and ACS, but also in patients with cardioembolic stroke and in patients with end-stage renal disease requiring dialysis [2,39,40]. Increased RP levels have also been reported in patients with severe thrombocytopenia and with essential thrombocythemia (ET); moreover, RP levels are a predictive factor for the recovery of bone marrow thrombopoietic activity in patients undergoing autologous bone marrow transplantation [21,41,42].

Some studies on patients affected by coronavirus disease 2019 (COVID-19) have detected significantly higher levels of RP, providing further evidence that reticulated platelets are clinically important in the inflammatory activity of the disease [9,43,44,45].

The platelet indices MPV and MPM RtcPlts (%), obtained by us with the Siemens ADVIA 2120 hematology system, are sensitive indicators of platelet activation, and can be of crucial importance for the interpretation of laboratory results and for the clinical decision-making process, but they do not discriminate the etiopathogenetic mechanism of platelet activation. PLT, MPV, MPM, MPC and RtcPlts (%), although modified in the pathological conditions evaluated, did not show a statistically significant difference (*p* > 0.05) between the medians obtained in patients with suspected sepsis at onset and patients with ACS at onset [20,24,38,46]. It is the inflammatory state that sepsis and ACS at onset have in common, probably linked to the rapid release of cytokines, which contributes to increasing the level of immature and reticulated platelets without allowing a differentiation between the clinical conditions [22,47] or providing any prognostic value.

Although the terms cross-linked platelets and immature platelets are used interchangeably, it is important to distinguish between the different indices because there is significant, but incomplete, overlap in the populations measured and a “method-dependent” expression of the results. The modest correlation (r^2^ = 0.38) and the lack of standardization and alignment between cross-linked platelet counts obtained with different analyzers result in highly variable results between clinical studies conducted with different methods, even within similar clinical populations [48,49,50,51]. The analytical variability of different automated hematology analyzers is related to the fluorescent dye used for RNA; to the measurement mode of scattered light (cell volume) and fluorescence intensity (in relation to RNA content); to the algorithm used to define the gating of cross-linked platelets; and to the ability to discriminate cross-linked platelets from interfering cells [52]. The RtcPlts (%) of Siemens analyzers is a calculated index that uses a moving threshold to separate mature platelets and cross-linked platelets and a refractive index histogram to calculate the threshold between cross-linked platelets and signals from hypochromic erythrocytes, lymphocytes, nuclear red blood cells (NRBCs) and possible interferents [38].

Clinical cutoffs and reference intervals of platelet indices and cross-linked platelets obtained with the Siemens ADVIA 2120 analyzer may be different from the values obtained with other analytical methods (Sysmex, Abbott, Mindray) and therefore are not interchangeable and must be specifically validated. In the literature, there is a lack of sufficient data on RtcPlts determined by Siemens ADVIA 2120 [53], hence the usefulness of a study that has evaluated and provided clinical cutoffs, in patients with suspected sepsis and ACS, of the RtcPlts parameter obtained with the Siemens ADVIA 2120 hematology analyzer [20,38].

## 5. Conclusions

Platelet indices and immature platelet counts can offer added value in the diagnosis of various pathological conditions, complementing the information provided by other biomarkers. Their use could be recommended to improve decision-making in the initial phase of a serious infection or ACS without generating additional costs, as they are determined during routine blood count testing. Consistent with the findings of numerous studies using different automated analyzers, the RtcPlts (%) parameter, determined with the Siemens ADVIA 2120, also proved to be a sensitive and specific marker, in the pathologies assessed, for signaling a state of inflammation. However, further studies with this parameter should be conducted to fully evaluate the role of RtcPlts in monitoring or prognosis rather than in the initial phase.

## Figures and Tables

**Table 1:** Evaluation of the absolute number of platelets, platelet indices and biomarkers in healthy subjects included in this study.

	PLT ×10^3^/µL	RtcPlts %	MPC g/dL	MPM pg/dL	MPV fL	WBC ×10^3^/µL	NG %	PSP pg/mL	Pct ng/mL	CRP mg/L	hs cTnI ng/L
**Number**	40	40	40	40	40	40	40	40	40	40	40
**Minimum**	155.70	0.14	18.72	1.64	6.30	4.06	44.55	18.9	0.01	0.18	8.0
**Maximum**	342.0	1.16	28.10	2.46	10.10	8.55	71.30	48.00	0.03	1.40	25.0
**Mean**	231.37	0.81	25.06	2.00	8.22	6.46	57.51	33.82	0.01	0.77	14.60
**95% CI**	216.38 to 246.34	0.72 to 0.89	24.44 to 25.66	1.93 to 2.06	7.93 to 8.49	6.04 to 6.88	55.9 to 59.63	31.36 to 36.28	0.011 to 0.02	0.67 to 0.86	13.23 to 15.96
**Median**	217.0	0.86	25.25	1.98	8.24	6.51	57.90	35.00	0.01	0.81	15.0
**95% CI**	213.38 to 238.66	0.78 to 0.96	24.03 to 26.36	1.91 to 2.09	7.81 to 8.66	5.85 to 7.11	54.18 to 60.20	31.50 to 36.90	0.01 to 0.02	0.61 to 0.90	12.67 to 15.0
**Normal Distribution**	0.024	0.030	0.040	0.877	1.0	0.584	0.257	0.235	<0.0001	0.202	0.009

**Table 2:** Evaluation of the absolute number of platelets, platelet indices, white blood cell counts and biomarkers of sepsis in subjects with suspected sepsis (s) at onset.

	PLT-s ×10^3^ µL	RtcPlts-s %	MPC-s g/dL	MPM-s pg	MPV-s fL	WBC-s ×10^3^/µL	NG-s %	Pct-s ng/mL	PSP-s pg/mL	CRP-s mg/L
**Number**	40	40	40	40	40	40	40	40	40	40
**Minimum**	36.0	1.68	21.10	1.72	8.30	1.40	23.3	0.48	407	12.60
**Maximum**	479.0	9.30	28.38	3.19	15.62	39.41	99.29	54.01	22,000	328.90
**Mean**	187.73	3.36	25.37	2.40	10.84	15.66	84.89	4.97	2921.15	97.49
**95% CI**	150.75 to 224.69	2.84 to 3.88	24.76 to 25.981	2.28 to 2.51	10.23 to 11.45	13.50 to 17.83	79.65 to 90.12	1.41 to 8.54	1507.78 to 4334.51	76.19 to 118.79
**Median**	154.00	2.85	25.25	2.37	10.32	15.56	87.15	1.78	1522.50	83.80
**95% CI**	111.01 to 236.25	2.53 to 3.45	24.71 to 26.21	2.25 to 2.54	9.93 to 11.07	14.46 to 16.63	85.02 to 92.16	1.29 to 2.34	1096.62 to 2297.06	71.10 to 114.44
**Normal Distribution**	0.0525	0.0028	0.2193	0.7917	0.0317	<0.0001	0.0002	<0.0001	<0.0001	0.0733

**Table 3:** Mann–Whitney test (independent samples) for the comparison of the absolute number of platelets, platelet indices, white blood cell counts and biomarkers of sepsis between control subjects and patients with suspected sepsis (s) at onset.

Control Group	Patients with Suspected Sepsis	Two-Tailed Probability
RtcPlts	RtcPlts-s	*p* < 0.0001
MPC	MPC-s	*p* = 0.3681
MPM	MPM-s	*p* < 0.0001
MPV	MPV-s	*p* < 0.0001
PLT	PLT-s	*p* = 0.0130
WBC	WBC-s	*p* < 0.0001
NG	NG-s	*p* < 0.0001
CRP	CRP-s	*p* < 0.0001
Pct	Pct-s	*p* < 0.0001
PSP	PSP-s	*p* < 0.0001

**Table 4:** Receiver operating characteristic curve (ROC) parameters for the absolute number of platelets and platelet indices in subjects with suspected sepsis (s) at onset.

Parameters	AUC	95% CI
PLT-s	0.66	0.52 to 0.79
MPV-s	0.93	0.88 to 0.98
MPM-s	0.84	0.74 to 0.92
MPC-s	0.56	0.42 to 0.68
RtcPlts-s	0.96	0.90 to 1.00

**Table 5:** Sensitivity, specificity and the best cutoff values obtained from the analysis of the ROC curves for the individual platelet indices in subjects with suspected sepsis (s) and in subjects with ACS (i).

Variable	Sensitivity	Specificity	Best Cutoff Values
MPV-s	87.5	87.5	9.1
MPV-i	77.5	90.0	9.2
MPC-s	27.5	92.5	27.1
MPC-i	82.5	40.0	24.03
MPM-s	65.0	95.0	2.27
MPM-i	65.0	90.0	2.21
RtcPlts-s	95.0	100	1.15
RtcPlts-i	95.0	100	1.16

**Table 6:** Evaluation of the absolute number of platelets, platelet indices and myocardial necrosis biomarker (hs cTnI) in subjects with ACS (i) at onset.

	PLT-i ×10^3^ µL	RtcPlts-i %	MPC-i g/dL	MPM-i pg	MPV-i fL	hs cTnI -i ng/L
**Number**	40	40	40	40	40	40
**Minimum**	36.0	1.21	18.70	1.79	7.80	217
**Maximum**	547.0	11.95	29.59	3.38	18.37	104,883
**Mean**	183.58	3.62	25.40	2.38	10.83	16,596.53
**95% CI**	147.01 to 220.13	2.68 to 4.56	24.60 to 26.19	2.26 to 2.49	10.04 to 11.61	6875.36 to 26,317.68
**Median**	177.50	2.38	25.55	2.29	10.23	3291.50
**95% CI**	127.37 to 240.66	2.11 to 3.20	24.70 to 26.79	2.19 to 2.43	9.36 to 11.07	1130.84 to 7334.81
**Normal Distribution**	0.0958	<0.0001	0.0446	0.1204	0.0024	<0.0001

**Table 7:** Mann–Whitney test (independent samples) for the comparison of the absolute number of platelets, platelet indices and hs cTnI between control subjects and patients with ACS (i) at onset.

Control Group	Patients with ACS	Two-Tailed Probability
RtcPlts	RtcPlts-i	*p* < 0.0001
MPC	MPC-i	*p* = 0.1447
MPM	MPM-i	*p* < 0.0001
MPV	MPV-i	*p* < 0.0001
PLT	PLT-i	*p* = 0.0189
hs cTnI	hs cTnI-i	*p* < 0.0001

**Table 8:** Receiver operating characteristic curve (ROC) parameters for absolute number of platelets and platelet indices in subjects with ACS (i) at onset.

Variable	AUC	95% CI
PLT-i	0.66	0.52 to 0.78
MPV-i	0.90	0.84 to 0.96
MPC-i	0.59	0.46 to 0.72
MPM-i	0.84	0.75 to 0.92
RtcPlts-i	0.95	0.89 to 1.00

**Table 9:** Mann–Whitney test (independent samples) for the comparison of the absolute number of platelets and platelet indices between patients with ACS (i) at onset and patients with suspected sepsis (s) at onset.

Patients with ACS	Patients with Suspected Sepsis	Two-Tailed Probability
RtcPlts-i	RtcPlts-s	*p* = 0.1321
MPC-i	MPC-s	*p* = 0.7727
MPM-i	MPM-s	*p* = 0.5252
MPV-i	MPV-s	*p* = 0.5832
PLT-i	PLT-s	*p* = 0.8398

## Data Availability

The data presented in this study are available on request from the corresponding author due to the privacy reasons (sensitive data).
